# Isotropic Brain MRI Reconstruction from Orthogonal Scans Using 3D Convolutional Neural Network

**DOI:** 10.3390/s24206639

**Published:** 2024-10-15

**Authors:** Jinsha Tian, Canjun Xiao, Hongjin Zhu

**Affiliations:** 1 School of Big Data and Artificial Intelligence, Chengdu Technological University, Chengdu 611730, China; canjunxiao@cdtu.edu.cn; 2 Digital Twin Laboratory, Chengdu Technological University, Chengdu 611730, China

**Keywords:** 3D convolutional neural network, isotropic reconstruction, magnetic resonance imaging, super-resolution, orthogonal scans

## Abstract

As an alternative to true isotropic 3D imaging, image super-resolution (SR) has been applied to reconstruct an isotropic 3D volume from multiple anisotropic scans. However, traditional SR methods struggle with inadequate performance, prolonged processing times, and the necessity for manual feature extraction. Motivated by the exceptional representational ability and automatic feature extraction of convolutional neural networks (CNNs), in this work, we present an end-to-end isotropic MRI reconstruction strategy based on deep learning. The proposed method is based on 3D convolutional neural networks (3D CNNs), which can effectively capture the 3D structural features of MRI volumes and accurately predict potential structure. In addition, the proposed method takes multiple orthogonal scans as input and thus enables the model to use more complementary information from different dimensions for precise inference. Experimental results show that the proposed algorithm achieves promising performance in terms of both quantitative and qualitative assessments. In addition, it can process a 3D volume with a size of 256 × 256 × 256 in less than 1 min with the support of an NVIDIA GeForce GTX 1080Ti GPU, which suggests that it is not only a quantitatively superior method but also a practical one.

## 1. Introduction

Magnetic resonance imaging (MRI) is a crucial and versatile medical imaging modality broadly used in clinical diagnosis and image-guided therapeutics. In principle, it utilizes the magnetic resonance phenomenon to collect data in the frequency domain and then generates images through inverse Fourier Transformation in situations where many sensors are involved in the process of generating MR images, including magnetic field generation, pulse signal excitation and reception, etc. [1,2]. In many MRI experiments, a basic consideration is how to achieve a good equilibrium between spatial resolution, signal-to-noise ratio (SNR), and acquisition time [3]. For instance, to reduce motion artifacts and improve the SNR of MR slices, many MRI scans are performed with relatively few slices and rather large slice thicknesses [4]. Consequently, most 3D MR volumes are collected as tomographic sets of image slices, which have higher resolution in imaging planes and lower resolution along the slice-select direction, causing anisotropic spatial resolution in 3D space [5]. This could be problematic for downstream tasks, such as computer-aided diagnosis [6], quantitative analysis, and visualization since the image will be missing more high-frequency information in the through-plane direction [4]. Therefore, it is much desired and beneficial to produce MRI data with 3D isotropic spatial resolution in practical applications.

An intuitive manner to acquire 3D isotropic and high spatial resolution MRI volumes is to improve the hardware devices of MRI scanners, e.g., higher magnetic fields, stronger and faster gradients, etc. However, these solutions are often expensive, require hardware upgrades, and are still subject to various complex factors, e.g., physical constraints, sensor performance, and system noise [4,5]. Conversely, to keep a similar SNR, reducing the voxel size to produce isotropic resolution requires averaging multiple acquisitions, thus increasing the imaging time and inflexibility in routine practice [7].

Another alternative is to enhance the resolution of magnetic resonance (MR) images along the through-plane direction using image post-processing techniques. One popular technique is termed super-resolution (SR) imaging, which aims at recovering a high-resolution (HR) image from one or more low-resolution (LR) images [8]. As a classical problem, image SR is still an active yet challenging hotspot in both the natural and medical image-processing communities [9]. In the literature, a variety of SR methods have been studied, such as interpolation methods [10], edge-based methods [11], modeling and reconstruction methods [12], and example-based learning methods [13], as well as dictionary learning methods [14,15]. In terms of isotropic MRI reconstruction, the methods in [4,16] adopted 3D block-based self-similarity learning and sparse representation to reconstruct HR volumes with isotropic resolution, which are essentially shallow learning techniques. These methods are inherently limited in SR performance because (1) finite additional information is used for solving the severely ill-posed inverse problem, and (2) the representational capacity of these models is inadequate for accurate SR inference [8,17].

In recent years, deep-learning-based [18] SR algorithms have been broadly and actively studied, and significantly facilitated the rapid development of various benchmark SR tasks. Owing to the powerful capacity of deep models to capture hierarchical features that map from LR space to HR space [8,19], recent methods based on deep learning [18] techniques have achieved great improvements in SR performance.

For natural images, a pioneering approach is SRCNN [20], which applies a three-layer convolutional neural network (CNN) [21] to super-resolve a single input image. Subsequently, a host of CNN-based SR models have been reported and some strategies have also been developed to improve the performance of deep models, such as increasing the model scale (including network parameter, depth as well as width) [22,23], residual learning [24,25], directly mapping from input LR images [26,27], adversarial training [28,29], etc. Other representative SR models include FC^2^N [30], SAN [31], SwinIR [32], ELAN [33], SRFormer [34], etc. In medical image processing, improving image quality and mitigating image degradation, e.g., image enhancement [35] and artifact removal [36], has been proposed to help promote the performance of subsequent processing and analysis. There are also some efforts to utilize deep learning technology to deal with medical image SR tasks, such as CSN [17], SERAN [37], DisC-Diff [38], Dual-ArbNet [39], McMRSR [40], etc. In the context of MRI isotropic reconstruction, however, there are relatively few works based on deep learning. One of the latest and possibly the most relevant works on this topic proposed to super-resolve brain MR images through the use of 3D CNNs [41], but it mainly focused on general image SR tasks, rather than isotropic MRI reconstruction.

Although significant progress has been made in deep learning in recent years, with advanced model architectures such as attention mechanisms [31,42,43] and Transformers [44,45,46] enhancing the performance of related tasks, these advanced structures are not very suitable for isotropic resolution reconstruction of MR images, as clinical applications have high requirements for fast inference. For 3D MR volumes, this issue is more prominent. Therefore, more advanced but complex architectures might not be suitable for isotropic MRI reconstruction in 3D scenarios.

**Figure 1:**
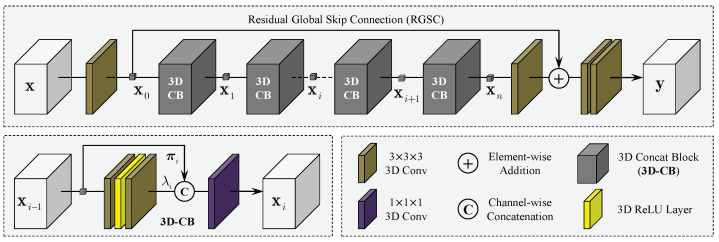
The overall network structure of the proposed 3D CNN model for isotropic MRI reconstruction. The feature extraction contains only one 3D conv layer and the image recovery part consists of two 3D conv layers. The nonlinear inference part is composed of several 3D concat blocks (3D-CBs), each of which is built with two 3D conv layers with one ReLU layer in the middle. Note that our 3D-CB is different from the residual block in [22].

In this work, we propose an isotropic reconstruction super-resolution network (isoSRN) to solve the problem of isotropic MRI reconstruction. For local feature fusion, our isoSRN extends weighted channel concatenation [30] and wide activation [47] to 3D space to capture sufficient 3D structural information, thereby contributing to accurate nonlinear SR inference. As with many other SR models [17,22,32,34], we introduce residual global skip connection (RGSC) to ease the training difficulty of the deep models. In addition, our isoSRN takes 3D orthogonal scans as input to make full use of orthogonal supplementary information, as in [4]. However, we utilize this information for deep feature learning instead of traditional sparse representation. The overall structure of our isoSRN is illustrated in Figure 1. With the strong representational capacity of 3D CNN models, our isoSRN can recover isotropic HR volumes more accurately. Extensive experiments show that our model is noticeably superior to other methods both quantitatively and qualitatively. The main contributions of this paper can be summarized as follows:

• We present a simple yet efficient 3D model for isotropic MRI reconstruction that utilizes multiple orthogonal LR volumes with anisotropic resolution to generate an isotropic HR volume.
• The proposed model is built upon simply extending wide activation [47] and weighted channel concatenation [30] to 3D space, which can promote 3D feature learning while maintaining efficient inference of the model.
• The proposed model is evaluated on several simulated and real MRI datasets, and it shows significant superiority to other compared methods in terms of both quantitative evaluations and qualitative analyses.

The rest of this work is arranged as follows. We first present the SR problem formulation in Section 2, and the details of the proposed model in Section 3. Then, the experimental results and analyses are given in Section 4. Finally, we conclude and discuss the whole paper in Section 6.

## 2. Problem Formulation

Image SR is usually formalized as an inverse problem with an ill-posed nature, which reconstructs an HR MR volume from one or more LR MR volumes according to the MRI imaging model. Given *V* LR observations $x_{1},$$x_{2},\ldots ,x_{V}\in R^{h\times w\times d}$ and their HR volume $y\in R^{H\times W\times D}$, the imaging model (or HR volume degradation model) can be usually formulated as:

(1) $$x_{v}=D_{v}M_{v}y+n_{v},\;\;\;v=1,2,\ldots ,V,$$

where $M_{v}$ represents the joint degradation operations on HR volume y to generate the *v*-th LR volume $x_{v}$, such as blurring and geometric transformations, etc., and $D_{v}$ is a downsampling operation that reduces the shape of HR volume to that of the LR volume $x_{v}$. $n_{v}$ usually denotes the Rician noise [48]. This formulation describes the task as a multiple-image SR (MISR) problem and it degrades to a single-image SR (SISR) problem when V=1. In the context of unsupervised learning, the SR image can be solved by optimizing the following loss:

(2) $$y^{*}=\underset{y}{\arg\min}\sum_{v=1}^{V}\left|\right|x_{v}-D_{v}M_{v}y{\left|\right|}_{2}^{2},$$

where $y^{*}$ is the prediction of HR image y. Directly optimizing Equation (2) typically leads to unstable solutions that require the utilization of appropriate regularization terms to well pose and stabilize the solution. Therefore, the general form of the target for image SR is usually expressed as:

(3) $$y^{*}=\underset{y}{\arg\min}\sum_{v=1}^{V}\left|\right|x_{v}-D_{v}M_{v}y{\left|\right|}_{2}^{2}+\gamma \phi \left(y\right),$$

where ϕ(y) is the regularization term associated with an image prior, and γ is a non-negative trade-off parameter that governs the compromise between the regularization term ϕ(y) and the fidelity term. However, the use of regularization still suffers from unstable solutions as it modifies implicitly the acceptable solution space without any guarantee of recovering realistic HR volumes [41], and how to choose a suitable regularization term and optimal trade-off parameter γ is practically difficult.

Compared with unsupervised learning, supervised learning techniques are superior in that they can reconstruct novel details that are not available in LR images. In a supervised context, the HR image y can be recovered with the following formulation, in which regularization is implicitly contained:

(4) $$y^{*}=\underset{y}{\arg\min}\sum_{v=1}^{V}||y-H_{v}U_{v}x_{v}{\left|\right|}_{2}^{2},$$

where $H_{v}$ and $U_{v}$ formalize the process of HR reconstruction and upsampling for $x_{v}$. For easier HR reconstruction and SR inference, we convert the MISR problem in Equation (4) to an SISR problem as follows: (i) upsample each LR volume $x_{v}$ by, e.g., interpolation: $x_{v}^{u}=U_{v}x_{v}$; (ii) fuse these upsampled volumes by simple element-wise average:

(5) $$x=\frac{1}{V}\sum_{v=1}^{V}x_{v}^{u}=\frac{1}{V}\sum_{v=1}^{V}U_{v}x_{v}.$$

As for HR reconstruction operation $H_{v}$, we assume that it behaves in the same manner for each $x_{v}$ (or the fused LR volume x) after the upsampling operation $U_{v}$. Therefore, let $H_{v}=H$ and then, Equation (4) can be rewritten as:

(6) $$y^{*}=\underset{y}{\arg\min}||y-{Hx||}_{2}^{2},$$

which evolves into the optimization objective of a typical SISR problem in the context of supervised learning. In the case of deep learning, H is usually modeled as an artificial neural network (ANN), for instance, a typical CNN architecture for complex nonlinear SR inference.

However, as far as image SR is concerned, it has been shown that $L_{1}$ loss has better convergence than $L_{2}$ loss, which is more beneficial to MR image SR tasks [17,22]. We, therefore, optimize a $L_{1}$ loss to solve Equation (6). Given a training dataset D consisting of |D| paired HR volumes y and the corresponding LR volumes x, the $L_{1}$ loss can is defined as:

(7) $$L_{1}\left(\theta \right)=\frac{1}{\left|D\right|}\sum_{i=1}^{\left|D\right|}∥y^{\left(i\right)}-F\left(x^{\left(i\right)};\theta \right){∥}_{1},$$

where F(·)=H represents the mapping function of the CNN structure from x to y, and θ denotes the parameter set of the network. $\hat{y}=F\left(x;\theta \right)$ is the estimate of sample label y.

## 3. Isotropic Super-Resolution Network

### 3.1. 3D Feature Extraction

Most current models based on CNNs adopt convolutions with zero-padding to keep the spatial size of the output features unchanged, such as [17,20,22,30,34,43,49], etc. In this paper, all the convolutional layers we discuss are with zero-filling and single stride.

**Figure 2:**
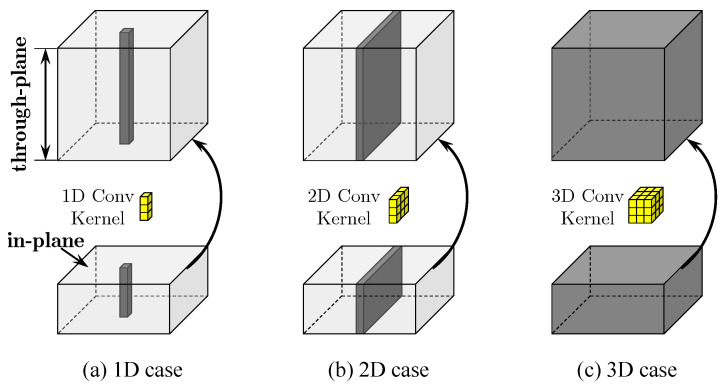
For isotropic MRI restoration, convolutional operations covering more dimensions can extract richer and more useful information. For instance, the information for inferring the feature map of the next layer, in the 1D case, is only from the dimension that requires to be scaled. However, in the 3D case (our work), the information in a 3D cube can be used to compute the feature map of the next layer.

In principle, each dimension of the LR volume $x_{v}$ may have anisotropic scaling factors to achieve isotropic reconstruction as the resolutions of all dimensions can be different from each other, while in practice, $x_{v}$ usually has the same high spatial resolution in the imaging plane and different low resolution in the through-plane direction. Therefore, the isotropic reconstruction of MR volumes is essentially a 1D SR problem that can even be solved through 1D CNN models. In this case, assume that $v^{t}\in R^{L\times C_{t}}$ denotes the intermediate feature tensor of the *t*-th layer with length *L* and channel number $C_{t}$, then, the feature tensor at the (t+1)-th layer is computed by:

(8) $$v_{m}^{t+1}\left(x\right)=\sigma [b_{m}^{t}+\sum_{n=1}^{C_{t}}\sum_{k=1}^{K_{l}}w_{nm}^{t}\left(k\right)v_{n}^{t}\left(\tilde{x}\right)],$$

where $m=1,\ldots ,C_{t+1}$ indexes over feature channels of $v^{t+1}$ and *n* indexes over those of $v^{t}$. $K_{l}$ indicates the length of 1D convolutional kernel $w^{t}\in R^{K_{l}\times C_{t}\times C_{t+1}}$. The parentheses are employed to indicate the spatial position of feature tensors and $\tilde{x}=x+k-K_{l}/2$. σ(·) represents a nonlinear function, e.g., a ReLU, and $b^{t}$ is the biases at the *t*-th layer whose length follows the number of output channels $C_{t+1}$. Therefore, only the information along the dimension to be upscaled is adopted to infer the feature map of the next layer, as demonstrated in Figure 2a. To use more information for inference, it is intuitive to extend the 1D convolution layer to a 3D convolution layer. Let $v^{t}\in R^{H\times W\times D\times C_{t}}$ represent the feature map at the *t*-th layer with $C_{t}$ channels, then, the feature map at the (t+1)-th layer is computed as follows in the 3D case:

(9) $$v_{m}^{t+1}\left(x,y,z\right)=\sigma \left[b_{m}^{t}+\psi_{m}^{t}\left(x,y,z\right)\right].$$

Here $\psi_{m}^{t}\left(x,y,z\right)$ is given by:

(10) $$\psi_{m}^{t}\left(x,y,z\right)=\sum_{n=1}^{C_{t}}\sum_{i=1}^{K_{h}}\sum_{j=1}^{K_{w}}\sum_{k=1}^{K_{d}}w_{nm}^{t}\left(i,j,k\right)v_{n}^{t}\left(\tilde{x},\tilde{y},\tilde{z}\right),$$

where *m* and *n* have the same meanings as in the 1D case, and $w^{t}\in R^{K_{h}\times K_{w}\times K_{d}\times C_{t}\times C_{t+1}}$ denotes the 3D convolutional kernel of the *t*-th layer with spatial size of $K_{h}\;\times \;K_{w}\;\times \;K_{d}$, and:

(11) $$\begin{array}{cc} \tilde{x} & =x+i-K_{h}/2,\\ \tilde{y} & =y+j-K_{w}/2,\\ \tilde{z} & =z+k-K_{d}/2. \end{array}$$

Therefore, in the case of 3D, in addition to exploring features with multiple channels, the network will also make full use of information in the 3D space to perform inference. Moreover, 3D convolutional kernels increase network parameters, obviously enlarging the representational capacity of the model.

### 3.2. Joint Linear Attention

Attention mechanisms are widely used to process the different components of an input signal distinctively. They decide the allocation of processing resources according to the information amount and importance of these signal components. Attention has been adopted extensively in deep learning and it shows the potential to improve model performance in many tasks such as image classification [50,51], object localisation [52], image restoration [49], etc. It is typically implemented by integrating a nonlinear function (e.g., a ReLU or sigmoid) with sequential operations [50], in the form of self-attention.

Unlike the above works, Zhao et al. [30] presented a novel joint linear attention mechanism for linear and nonlinear features in the network, which is proven to be conducive to the full mining of model representational capacity. However, they mainly aim at natural image SR tasks in 2D. In this paper, we extend the joint linear attention to 3D space and use it for the isotropic reconstruction of MRI volumes. Let […] denote the operation of channel concatenation, then a channel concatenation block in 3D space (3D CB) can be formulated as the following:

(12) $$x_{i}=L\left([\pi_{i}x_{i-1},\lambda_{i}H\left(x_{i-1}\right)]\right),$$

where L(·) corresponds to the 1×1×1 convolutional layer at the end of a CB, as shown in Figure 1. Furthermore, H(·) represents the function of the *nonlinear mapping* branch, and $\pi_{i}$ and $\lambda_{i}$ represent the weighting factors of the *identity* branch and nonlinear mapping branch, respectively. Let t=i−1 and $u^{t}=H\left(x_{i-1}\right)$, and we remove the *spatial* dimensions of the 1 × 1 × 1 convolutional kernel $w^{t}\in R^{1\times 1\times 1\times 2C_{t}\times C_{t+1}}$ for ease of representation. Then, we can follow the convention in Section 3.1 to reformulate the 3D weighted channel concatenation:

(13) $$\begin{array}{cc} x_{m}^{t+1}\left(x,y,z\right) & =\sum_{n=1}^{C_{t}}\pi_{t+1}w_{nm}^{t}x_{n}^{t}\left(x,y,z\right)\\ \; & +\sum_{n=1}^{C_{t}}\lambda_{t+1}w_{(n+C_{t})m}^{t}u_{n}^{t}\left(x,y,z\right). \end{array}$$

Since the overall process fuses *identity* mapping and *nonlinear mapping*, and no activation is attached to the 1 × 1 × 1 convolutional layer, it can be viewed as a joint linear attention of the linear and nonlinear features. In addition, owing to the learnability of w, λ and π, when (i) $w_{nn}^{t}=w_{(n+C_{t})n}^{t}=1$ for $n=1,2,\ldots ,C_{t}$ and the other elements in $w^{t}$ are 0, and (ii) $\pi_{t+1}=\lambda_{t+1}=1$, then the weighted channel concatenation actually degrades to the residual connection. In this case, if π and λ are learnable in the 2D case, it degrades to adaptive residual learning [30].

### 3.3. Network Architecture

The overall network architecture is shown in Figure 1. Similar to many previous SR networks, such as [17,22,30] etc., the proposed isoSRN is modularized and consists of three phases: feature extraction, non-linear mapping, and image reconstruction.

Feature extraction is achieved by a 3 × 3 × 3 convolutional layer, which is used to simulate the dense patch extraction in many conventional methods such as sparse representation and dictionary learning. To protect low-level visual features, it is usually embedded in the network without a nonlinear activation function. The nonlinear inference part of the proposed isoSRN model consists of a group of cascaded 3D CB blocks, whose internal structure is shown in Figure 1. For the nonlinear branch of a CB block, the sequential operations of Conv-ReLU-Conv are adopted with wide activation [47]. Suppose there are *n* CB blocks in the network and the mapping function of the *i*-th block is denoted as $B_{i}\left(\cdot \right)$, then the entire nonlinear mapping process of our isoSRN can be iteratively formulated as:

(14) $$x_{n}=B_{n}\left(x_{n-1}\right)=B_{n}\left(B_{n-1}\left(\cdots \left(B_{1}\left(x_{0}\right)\right)\cdots \right)\right),$$

where $x_{0}$ denotes the extracted feature by the first 3 × 3 × 3 convolutional layer, as illustrated in Figure 1, and $x_{n}$ implies the deep features of the network. Next, the shallow features $x_{0}$ and deep features $x_{n}$ are integrated by a commonly-used residual connection: $x_{0}+x_{n}$. Image reconstruction is implemented by two 3 × 3 × 3 convolutional layers. The first convolutional layer is used for further fusion of shallow and deep features and the second one is employed to map these features into HR image space, as shown in Figure 1. We also demonstrate the architecture of our model in Algorithm 1.   
**Algorithm 1:** The isoSRN Model for Isotropic MRI Reconstruction
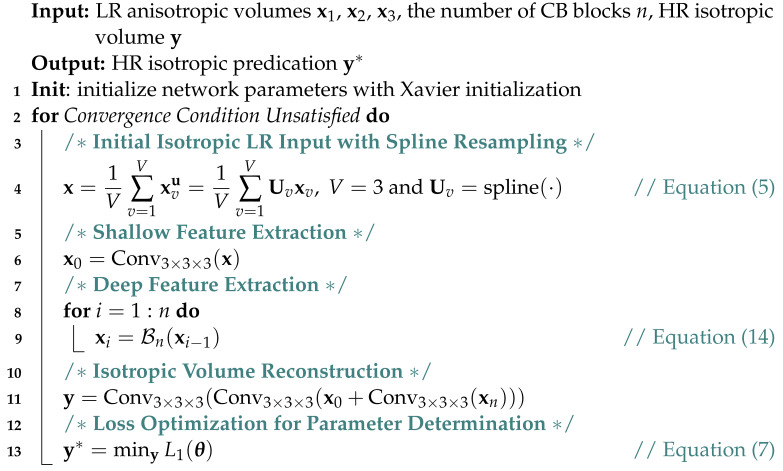


### 3.4. Network Scale

Network scale usually refers to the depth, width, and number of parameters of the network. In general, a larger network scale indicates a stronger representational ability of the model, as well as better performance. In particular, the depth and number of parameters have a significant impact on the performance of the model. The depth of a deep network is typically defined as the longest path from the input to output [17]. As for the proposed isoSRN, the depth can be formulated according to Figure 1:

(15) D=3n+4,

where *n* denotes the number of 3D-CBs. We set n=16 in our implementation, and *D* therefore is 52. It can be seen that *D* is independent of the scaling factor since the nonlinear inference is in HR image space. This increases the computational effort of the model, but the advantage is that it is easy to implement and can deal with arbitrary scaling factors, including fractional factors. Conversely, the proposed isoSRN takes ≈ 3.63 M learnable parameters according to Figure 1 and the description in Section 4.1. This is a moderate amount of parameters, which is also amenable to practical deployment.

## 4. Experimental Results

### 4.1. Datasets and Implementation Details

We randomly chose 155 pair structural MR volumes from the HCP dataset (https://www.humanconnectome.org/, accessed on 5 September 2022) [53] (including both T1 and T2 data). These volumes were collected with 0.7 mm × 0.7 mm × 0.7 mm isotropic resolution and matrix size of 260  × 311 × 260. We divided these 155 volumes into 100 training samples, 50 testing samples, and 5 fast validation samples. To verify the ability of the proposed isoSRN model to process other MR data, we collected three other datasets: Sim-H, Sim-P, and Set7, which contain two, four, and seven volumes, respectively. The detailed information of these datasets is shown in Table 1. Note that Sim-H and Sim-P are generated from simulated data from BrainWeb (https://brainweb.bic.mni.mcgill.ca/brainweb/, accessed on 12 August 2022) [54], while Set7 consists of in vivo data acquired using a 3T GE scanner with a T1-3D FSPGR sequence (TR/TE = 5.936 ms/1.956 ms and flip angle [FA] = 9^∘^, matrix size = 256 × 256, and field of view [FOV] = 25.6 × 25.6 cm^2^, slice thickness = 1 mm).

The kernel size of the proposed isoSRN follows the annotation of Figure 1, and we set the number of 3D CB blocks to 16. The number of feature maps is set to 32, which is magnified by a factor of 4 in the wide activation [47]. The learnable weighting factors $\lambda_{i}$ and $\pi_{i}$ are initialized to 1.0 before model training. We extract 24 × 24 × 24 cubes from LR volumes with their corresponding HR cubes from HR volumes to train the model. Data augmentation is completed by flipping up and down, left and right, and back and forth. We set the batch size to 8 for fast training. The $L_{1}$ loss function in Equation (7) is minimized using the Adam optimizer [55] by setting $\beta_{1}=0.9$, $\beta_{2}=0.999$ and $\epsilon =10^{-8}$. The learning rate is initialized as $2\;\times \;10^{-4}$ and halved at every $10^{5}$ iterations, with $4\;\times \;10^{5}$ iterations in total.

### 4.2. Training Example Generation

**Figure 3:**
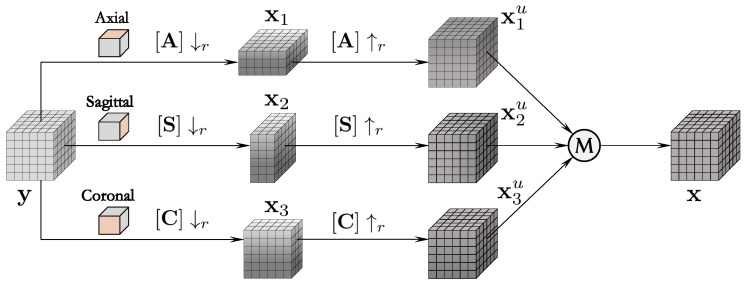
Sample generation. y represents a 3D HR volume with isotropic resolution and x denotes the corresponding LR volume with isotropic resolution. M is an operation of element-wise averaging. ${\left[d\right]↓}_{r}$ and ${\left[d\right]↑}_{r}$ stand for *r*-fold downsampling and upsampling along the dimension d, which could be A (Axial), S (Sagittal), or C (Coronal).

Given a HR volume y with isotropic resolution, we generate the corresponding LR volumes $x_{v},vs.=1,\ldots ,V$, according to the procedure shown in Figure 3. We utilize three orthogonal scans for isotropic MRI reconstruction in this paper, therefore, V=3 here. Firstly, we downsample the isotropic HR volume y along three orthogonal directions to generate three LR volumes with anisotropic resolution, which simulates three orthogonal scans. For downsampling, we apply spline interpolation to fuse multiple slices into one single slice (weighted average). This simulates the partial volume effect (PVE) that increases as the slice thickness increases.

Subsequently, we upscale these anisotropic volumes to the expected size by the same interpolation. This implements the upsampling operation $U_{v}$ in Equation (5). Lastly, these upsampled LR volumes $x_{v}^{u}$ are fused into a single LR volume x by simple element-wise averaging corresponding to the operation “M” in Figure 3 and Equation (5). In this manner, the LR volume x and HR volume y constitute a pair of samples for model training.

### 4.3. Evaluations of the Proposed Method

In this subsection, we evaluate the proposed isoSRN model on both simulated and clinical MR volumes (T1w and T2w) to verify its effectiveness in terms of various scenarios including scaling factor, noise level, pathology, and the number of orthogonal scans, as well as testing on in vivo MR data. We compare our isoSRN model with two traditional methods and a CNN-based method, namely, CubeAvg [4], NLM [56], and SRCNN-3D [20], respectively, for a comprehensive evaluation.

For a quantitative evaluation, we employ peak signal-to-noise ratio (PSNR), structural similarity index measurement (SSIM) [57], and image sharpness [58] as evaluation metrics. We also utilize the geometric self-ensemble [22] to further boost model performance, which is represented as isoSRN+.

#### 4.3.1. Slice Thickness

In this work, anisotropic LR images are generated by fusing multiple adjacent slices into a single slice in a weighted-average manner. This process is used to simulate the PVE in anisotropic acquisition. Typically, it gets stronger as the slice thickness increases [4], which corresponds to the scaling factor of image SR. To verify the effectiveness of the proposed isoSRN model under different MR image types and scaling factors, we experiment on both T1 and T2 data with six scaling factors: ×2∼×7. This indicates that we are going to reconstruct isotropic HR volumes with voxel size 0.7 mm × 0.7 mm × 0.7 mm from three orthogonal anisotropic scans with slice thicknesses of 1.4 mm, 2.1 mm, 2.8 mm, 3.5 mm, 4.2 mm, and 4.9 mm, respectively.

Table 2 exhibits the quantitative comparison between these methods in terms of PSNR and SSIM. It can be seen that our isoSRN model outperforms other methods by a large margin for all scaling factors. For instance, compared with CubeAvg [4], the PSNR value of isoSRN^+^ on T1w MR data for SR × 2 is 11.68 dB higher. For all the compared methods, we can observe that PSNR/SSIM values decrease as the scaling factor increases. Nevertheless, for SR × 7 on T2w data, our isoSRN+ still obtains PSNR/SSIM gains of 7.67 dB/0.0851.

**Figure 4:**
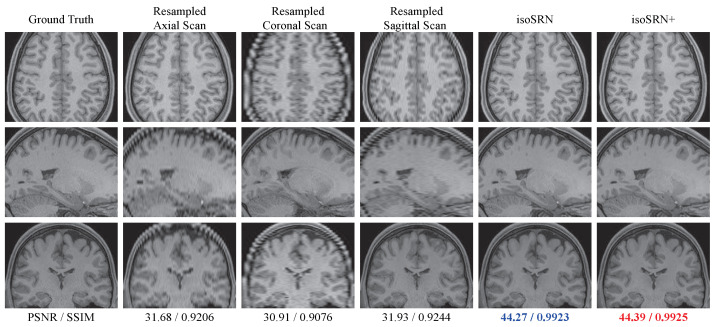
The visualization of the proposed isoSRN on a T1 volume in HCPtest (SR × 5, slice thickness = 3.5 mm). Top to bottom: Axial, Coronal, and Sagittal views. The 2nd to 4th columns are resampled scans through spline interpolation. The best result is marked in **red**, and the second-best is marked in **blue**.

Figure 4 displays three orthogonal slices of the reconstructed T1w MR volume in the HCPtest dataset when the slice thickness is 3.5 mm, i.e., SR × 5. For display purposes, the anisotropic scans are resampled to the same size as the HR isotropic volume via spline interpolation. As can be seen, the slices generated by resampling are heavily blurred and many details are lost in the 2nd∼4th columns in Figure 4, due to the PVE. However, our isoSRN and isoSRN^+^ can produce pleasing visual results with isotropic and high resolution.

#### 4.3.2. Noise Power

In MRI, raw data is intrinsically complex-valued and usually corrupted with zero mean Gaussian noise with equal variance [59]. After inverse Fourier transformation, MR magnitude data has been shown to be Rician distributed [58]. Since Rician noise is commonly found in MR images, it is important to study the influence of noise on model performance. To this end, we simply add (Note that Rician noise is not additive but data-dependent [58], here “add” just means to make the MR magnitude data be Rician-distributed). Rician noise with σ=0,5,10,15,20, and 25 to anisotropic scans $x_{v}$, and train the models with noise-free labels. Therefore, the proposed isoSRN model still works in an end-to-end manner without additional denoising steps when dealing with noisy samples.

Table 3 shows the reconstruction accuracy of the compared methods on the simulated dataset Sim-H, which contains two volumes representing T1w and T2w data of a healthy subject in the BrainWeb dataset. It can be observed that the proposed isoSRN (or isoSRN+) model greatly surpasses the traditional methods at all noise levels. For instance, in the case of Rician noise level σ=5 and slice thickness = 3.5 mm (SR × 5), the isoSRN+ achieves a significant PSNR/SSIM improvement of 10.79 dB/0.093 relative to CubeAvg. Even with a large Rician noise power, e.g., σ= 20 or 25, the superiority of the proposed isoSRN model over other methods is still remarkable. Another observation in Table 3 is that the reconstruction accuracy of all methods decreases as the noise power increases. This is easy to understand because the increased noise power makes image degradation more serious and the reconstruction problem of MRI images more difficult.

#### 4.3.3. Pathology

To study the reconstruction performance of the proposed isoSRN model on pathologic scans, we collected a multiple sclerosis (MS) dataset Sim-P extracted from the BrainWeb dataset, which contains four volumes with normal, mild, moderate, and severe MS, respectively.

The quantitative performance of the compared methods on this dataset is shown in Table 4. Note that these results are obtained by testing the model trained with HCP data directly on the Sim-P dataset. We can observe that our isoSRN^+^ model still outperforms traditional methods significantly, e.g., for SR × 2 on T1w data, the PSNR of the isoSRN^+^ is 12.65 dB higher than that of the CubeAvg [4], which is a large increment. Even with a large slice thickness of 4.9 mm, the increment still reaches 10.60 dB. Similar results can also be observed in the T2w data.

**Figure 5:**
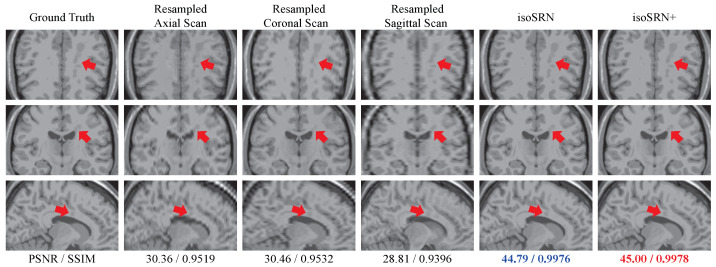
Top to bottom: Axial, Coronal, and Sagittal views of a simulated multiple sclerosis T1 volume in Sim-P (SR × 4, slice thickness = 2.8 mm). The best result is marked in **red**, and the second-best is marked in **blue**.

Figure 5 shows the reconstruction results of the proposed model over the simulated severe MS T1w volume, accompanied by the anisotropic scans. The red arrows indicate the locations of multiple sclerosis in different views. Similar to other structures in the image, the sclerosis can easily become blurred and obscured in the thick-slice scans, making it difficult to distinguish from other structures. However, in the results using our models, these lesions are satisfactorily recovered with an appearance close to the ground truth, as shown in the last two columns of Figure 5.

#### 4.3.4. The Number of Input Scans

**Figure 6:**
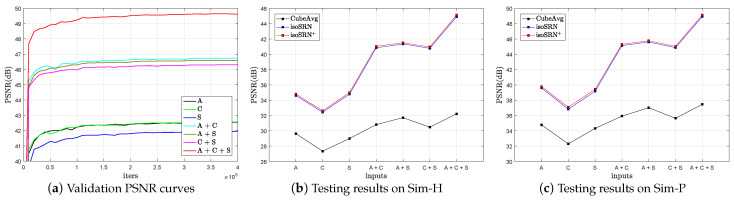
The influence of the number of input anisotropic LR scans (T1, SR × 3). When there is only one input scan, e.g., A, C, or S, CubeAvg is equivalent to simple spline interpolation. **A**: Axial scan; **C**: Coronal scan; **S**: Sagittal scan.

Similar to [4,16], in this section, we also investigate the influence of the number of input scans on the performance of the proposed method. To this end, we train the proposed model with different combinations of three orthogonal scans. When the number of input scans is equal to 1, the input x of the model is equivalent to the simple spline interpolation of the original LR scans. Once the models are well-trained, they are tested on two simulated datasets, Sim-H and Sim-P (T1w), with scaling factor SR × 3. Figure 6a shows the validation curves when training the models with different numbers of input scans. It can be seen that the model converges rapidly and stably in all cases. An obvious observation is that model performance remains basically at the same level when the input scan number is the same (1 or 2), but it is improved significantly when the number of input scans increases. We can also make the same observation about the testing results shown in Figure 6b,c.

It is worth noting that, unlike traditional optimization-based methods (e.g., [4,16], the time efficiency of our method is not affected by the number of inputs due to the end-to-end feature. For an input with a size of 260 × 311 × 260, the running time of the isoSRN model is about 40 s with the support of a single NVIDIA GeForce GTX 1080 Ti GPU. However, the method described in [4] takes more than 10 min to perform a complete reconstruction from three orthogonal scans.

### 4.4. Comparison with Other Methods

To fully compare the proposed method with other advanced methods, we introduce two other comparative models: ReCNN [41] and VDSR3D [23]. The former is a residual network with 10 Conv+ReLU units that uses a framework similar to ours. The latter is an extension of VDSR [23] based on the framework of the proposed solution. We introduce these methods for comprehensive comparison since they are easy to reproduce and have a similar scale of parameters to our isoSRN.

#### 4.4.1. Evaluation on In Vivo Data

To verify the generalization of our isoSRN to other data, we also present the comparative results on an in vivo dataset, Set7, as shown in Table 5. Although Set7 is derived from imaging hardware, the subjects, parameters, and environment are totally different from the HCP training samples [53], our isoSRN still works well and surpasses the compared methods by a large margin. For instance, the proposed isoSRN and isoSRN+ produce 10.11 dB and 10.23 dB PSNR gains relative to the baseline CubeAvg [4] for SR × 2. Even for large scaling factors, e.g., SR × 7, the performance increment is still up to 7.44 dB and 7.56 dB, respectively. This fully illustrates the good adaptability of the proposed method to in vivo data.

**Figure 7:**
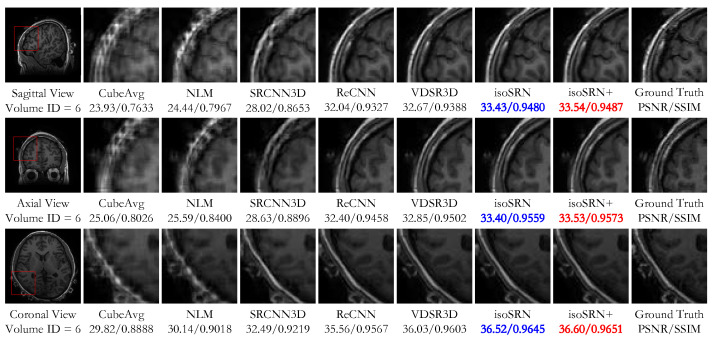
Visual comparison between the compared methods on the in vivo dataset Set7 for SR × 7. The best result is marked in **red**, and the second-best is marked in **blue**. Top to bottom: Sagittal, Axial, and Coronal views.

**Figure 8:**
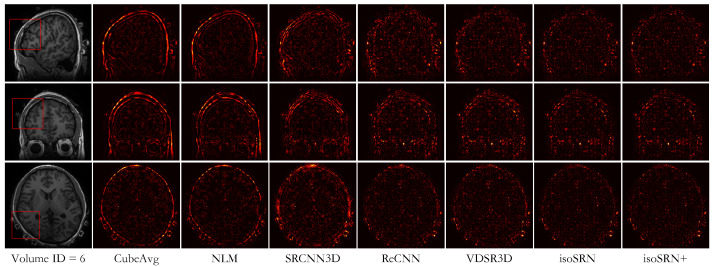
Visualization of the residuals between reconstruction results of different methods and the ground truth, corresponding to Figure 7. Higher pixel intensity indicates a larger difference between the reconstruction and ground truth (SR × 7). Top to bottom: Sagittal, Axial, and Coronal views.

Figure 7 displays the visual comparison between these methods on an in vivo volume from Set7, for SR × 7. We can clearly see the remarkable visual superiority of deep learning methods to traditional methods. Meanwhile, Figure 8 shows the residuals between the results of the compared methods and the ground truth, where we can observe that our proposed models present a better approximation to the ground truth. This conclusion is also demonstrated by the quantitative results annotated below the clipped images in Figure 7.

#### 4.4.2. Running Time

**Figure 9:**
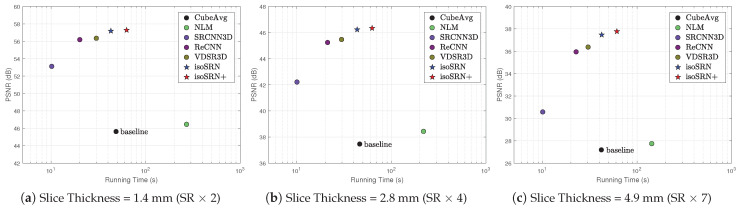
Inference time versus model performance on HCPtest dataset (T1). Note that except CubeAvg [4], we only collect the inference time for the mapping from x to y and exclude the time for fusing $x_{v}\left(v=1,\;2,\;3\right)$ to x.

Our solution for isotropic resolution MRI reconstruction is an end-to-end mapping with three orthogonal anisotropic scans as inputs. Therefore, the running time of the entire model involves two parts: fusion of isotropic LR volume x from three anisotropic volumes ${\left\{x_{v}\right\}}_{v=1}^{3}$ and SR inference of isotropic volumes y. Furthermore, the first part actually corresponds to the baseline CubeAvg [4]. Figure 9 compares the tradeoff of these methods on HCPtest (T1) for three scaling factors. The results are collected with an Omnisky workstation equipped with 64 GB memory, two Intel Xeon E5-2630 CPUs (2.20 GHz), and four NVIDIA GeForce GTX 1080 Ti GPUs. Note that we only consider the running time of SR inference for NLM [56], SRCNN3D [20], ReCNN [41], VDSR3D [23], and our isoSRN.

It can be seen that although our isoSRN runs slightly slower than other deep-learning-based methods, it is noticeably faster than the traditional method NLM [56]. Because our isoSRN performs significantly better than other compared methods (see Table 2, Table 3, Table 4 and Table 5), it provides a better compromise between model performance and running efficiency. Moreover, we can also see that as the input gets larger, the efficiency advantage of end-to-end mapping over traditional methods becomes more obvious. It can be also seen that the running efficiency of SR inference is independent of SR scaling factors.

## 5. Discussion and Future Work

### 5.1. Comparative Methods

In the proposed method, we need to acquire three orthogonal LR scans and upsample them with spline interpolation; thus, it is hard to make a fair comparison between our isoSRN model and LRTV [60], which recovers isotropic HR volumes directly from 3D LR volumes. Furthermore, we cannot compare our approach with [4,16] because the authors did not release the source code of their methods. Intuitively, however, the proposed isoSRN model can perform better than [4,16] in that it performs end-to-end mappings with some operations in the pipeline of [4,16] implicitly included in the optimization, e.g., patch extraction and image recovery. In addition, the techniques utilized in [4,16] can be substantially viewed as shallow learning models [20] that have limited representational capacity. Conversely, due to iterative optimization in the implementation, the methods described in [4,16] should be much slower than our model.

### 5.2. Multiple and Fractional Scales

In real MRI scenes, the ratio of in-plane resolution to that of slice-select direction can be arbitrary, even fractional. How then does a single CNN model deal with this case? In fact, the CNN can be “taught” to do this by simply fusing the corresponding training samples into the training set [9,23]. In the pipeline of our isoSRN model, this is easy to implement because we upsample LR volumes with spline interpolation before feeding them into the network and conduct nonlinear inference in the HR image space. In image SR, performing nonlinear inference in LR feature space helps to improve the training and inferring efficiency, but it is inconvenient in the case of multiple and fractional scaling factors. In this regard, the element-wise average (i.e., “M” in Figure 3 and Equation (5)) used to fuse multiple orthogonal scans is more friendly to practical applications due to its simplicity and easy implementation.

### 5.3. Generalization to Other Data

As described in Section 4.1, the proposed model is trained with 100 HCP samples [53], while tested on four datasets as shown in Table 1. Except in the case of HCPtest [53], the datasets have very different imaging conditions from those of the training data. In Section 4, however, we can observe a consistent improvement in the performance of the proposed isoSRN, which demonstrates its favorable generalization to different types of datasets. This also reveals the great representational capacity of deep models, and the ease of use in practical applications when considering the convenience of automatic feature extraction.

### 5.4. Extension to Real-World Scenarios

Three orthogonal scans of the subject are required to obtain three anisotropic volumes $x_{v}\left(v=1,\;2,\;3\right)$ when deploying our framework in practical applications. The critical issue is how to fuse $x_{v}$ accurately to generate LR volume x with isotropic resolution for SR inference. However, due to the discrepancies in brightness, contrast, and displacement between ${\left\{x_{v}\right\}}_{v=1}^{3}$, it is a challenging problem in itself to accurately fuse these LR volumes. An intuitive solution is to calibrate and correct these LR volumes before element-wise averaging but possibly with low accuracy. Another manner may be building a multi-branch network that takes anisotropic LR volumes ${\left\{x_{v}\right\}}_{v=1}^{3}$ as inputs and generates the fused LR volume x, or directly produces HR volume y. In future work, we will delve into these scenarios and drive the practical deployment of the solution.

## 6. Conclusions

This paper presents an end-to-end method based on deep 3D CNNs for reconstructing an HR volume with isotropic resolution from multiple anisotropic LR acquisitions. With the effective characterization of structural features in 3D space by deep 3D CNNs and the complementary information provided by orthogonal scans, the proposed isoSRN can surpass traditional methods by a large margin, as shown by our qualitative and quantitative experiments. Moreover, because it works in an end-to-end manner and does not require manual feature extraction, it is more practical and clinically flexible than traditional methods like NLM [56], sparse representation, and dictionary learning [4,16].

In this study, we presented a preliminary study of the application of CNNs in MRI fusion and isotropic reconstruction. Similar methods can be extended to other MRI applications, such as fMRI, dynamic cine MRI, etc., through high-dimensional convolutional models to capture spatio-temporal features, promoting subsequent diagnosis and computer-aided analysis of these MRI data.

## Figures and Tables

**Table 1:** Details of the testing datasets used in this work. Note that these data are collected with isotropic resolution. # denotes the volume number of a dataset.

Datasets	Mode	Dims	# Volumes	Voxel Size	Source
HCPtest [53]	T1/T2	260 × 311 × 260	50	0.7 mm	HCP
Sim-H [54]	T1/T2	217 × 181 × 181	2	1.0 mm	Brainweb
Sim-P [54]	T1/T2	217 × 181 × 181	4	1.0 mm	Brainweb
Set7 (Collected)	T1	256 × 256 × 154	7	1.0 mm	in vivo

**Table 2:** Performance comparison on **HCPtest** dataset in terms of different scaling factors (×2∼×7). The largest values are marked in red, and the second-largest are marked in blue (PSNR (dB)/SSIM). Both T1- and T2-weighted MR volumes are included here.

Methods	Type	SR × 2	SR × 3	SR × 4	SR × 5	SR × 6	SR × 7
CubeAvg [4]	T1	45.61/0.9937	40.19/0.9803	37.45/0.9648	35.66/0.9482	34.22/0.9292	33.29/0.9130
NLM [56]		46.44/0.9949	40.95/0.9834	38.42/0.9716	36.35/0.9559	34.85/0.9329	33.72/0.9220
SRCNN3D [20]		53.10/0.9986	45.66/0.9928	42.20/0.9850	39.87/0.9754	38.14/0.9646	36.86/0.9538
isoSRN [Ours]		57.19/0.9993	49.35/0.9965	46.23/0.9933	44.33/0.9901	42.84/0.9866	41.78/0.9834
isoSRN^+^ [Ours]		57.29/0.9993	49.46/0.9966	46.34/0.9934	44.44/0.9903	42.95/0.9869	41.92/0.9838
CubeAvg [4]	T2	41.01/0.9920	35.58/0.9736	33.10/0.9543	31.53/0.9345	30.25/0.9121	29.41/0.8932
NLM [56]		41.77/0.9935	36.36/0.9784	33.99/0.9632	32.11/0.9437	30.77/0.9235	29.77/0.9036
SRCNN3D [20]		49.29/0.9985	40.91/0.9909	37.23/0.9800	34.86/0.9662	33.12/0.9507	31.94/0.9360
isoSRN [Ours]		55.02/0.9994	45.51/0.9961	41.91/0.9919	39.77/0.9875	38.13/0.9825	36.91/0.9776
isoSRN^+^ [Ours]		55.19/0.9994	45.66/0.9962	42.05/0.9921	39.92/0.9878	38.28/0.9829	37.08/0.9783

**Table 3:** Performance comparison on the **Sim-H** dataset in terms of Rician noise power (σ=0∼25). The largest values are marked in red, and the second-largest are marked in blue (PSNR (dB)/SSIM).

Rician Noise	Scale	σ=0	σ=5	σ=10	σ=15	σ=20	σ=25
CubeAvg [4]	SR × 5	26.50/0.9206	26.45/0.8979	26.30/0.8546	26.07/0.8184	25.77/0.7902	25.43/0.7668
NLM [56]		27.21/0.9352	27.17/0.9142	27.05/0.8739	26.86/0.8413	26.62/0.8166	26.33/0.7966
isoSRN [Ours]		37.80/0.9923	36.86/0.9899	36.18/0.9882	35.67/0.9856	35.21/0.9828	34.81/0.9810
isoSRN^+^ [Ours]		38.02/0.9929	37.24/0.9909	36.65/0.9894	36.15/0.9873	35.70/0.9841	35.29/0.9829
CubeAvg [4]	SR × 7	23.68/0.8500	23.59/0.8294	23.52/0.7899	23.41/0.7567	23.26/0.7307	23.08/0.7089
NLM [56]		24.07/0.8668	24.05/0.8477	24.00/0.8127	23.92/0.7839	23.81/0.7615	23.67/0.7434
isoSRN [Ours]		33.43/0.9789	32.75/0.9760	32.34/0.9729	32.05/0.9688	31.81/0.9666	31.51/0.9627
isoSRN^+^ [Ours]		33.68/0.9809	33.24/0.9788	32.83/0.9755	32.60/0.9722	32.33/0.9701	32.05/0.9662

**Table 4:** Quantitative performance of the compared methods on the simulated pathologic dataset **Sim-P**, which contains four MR volumes with four different degrees of multiple sclerosis and two image types T1w and T2w. The largest values are marked in red, and the second-largest are marked in blue (PSNR (dB)/SSIM).

Methods	Type	SR × 2	SR × 3	SR × 4	SR × 5	SR × 6	SR × 7
CubeAvg [4]	T1	44.10/0.9977	37.46/0.9901	33.44/0.9760	30.65/0.9557	28.75/0.9329	27.17/0.9043
NLM [56]		44.39/0.9980	38.04/0.9921	34.32/0.9816	31.52/0.9642	29.55/0.9444	27.73/0.9151
SRCNN3D [20]		51.23/0.9995	43.47/0.9970	38.31/0.9903	35.11/0.9799	32.71/0.9650	30.56/0.9421
isoSRN [Ours]		56.58/0.9998	48.93/0.9990	44.79/0.9977	41.91/0.9953	39.71/0.9921	37.47/0.9868
isoSRN^+^ [Ours]		56.75/0.9998	49.14/0.9991	45.00/0.9978	42.12/0.9956	39.96/0.9925	37.77/0.9878
CubeAvg [4]	T2	44.10/0.9977	37.46/0.9901	33.44/0.9760	30.65/0.9557	28.75/0.9329	27.17/0.9043
NLM [56]		44.39/0.9980	38.04/0.9921	34.32/0.9816	31.52/0.9642	29.55/0.9444	27.73/0.9151
SRCNN3D [20]		50.97/0.9995	43.11/0.9969	38.24/0.9907	34.96/0.9789	32.67/0.9638	30.39/0.9407
isoSRN [Ours]		55.98/0.9998	48.54/0.9991	44.33/0.9975	41.49/0.9950	39.31/0.9916	37.01/0.9858
isoSRN^+^ [Ours]		56.18/0.9998	48.75/0.9991	44.56/0.9976	41.71/0.9953	39.57/0.9920	37.31/0.9867

**Table 5:** Performance comparison on Set7 dataset in terms of different scaling factors (×2∼×7). The best values are marked in red, and the second-best are marked in blue (PSNR (dB)/SSIM).

Methods	Type	SR × 2	SR × 3	SR × 4	SR × 5	SR × 6	SR × 7
CubeAvg [4]	T1	43.12/0.9927	38.23/0.9797	35.75/0.9656	34.07/0.9499	32.84/0.9334	32.07/0.9200
NLM [56]		44.62/0.9946	39.03/0.9825	36.65/0.9716	34.63/0.9559	33.34/0.9414	32.29/0.9266
SRCNN3D [20]		49.58/0.9980	42.96/0.9913	39.75/0.9828	37.55/0.9720	35.93/0.9599	34.79/0.9481
ReCNN [41]		52.46/0.9988	46.18/0.9953	43.22/0.9913	41.18/0.9866	39.59/0.9809	38.46/0.9752
VDSR3D [23]		52.42/0.9988	46.38/0.9955	43.50/0.9917	41.52/0.9875	39.95/0.9822	38.88/0.9773
isoSRN [Ours]		53.23/0.9990	46.76/0.9958	43.90/0.9924	42.04/0.9887	40.56/0.9843	39.51/0.9802
isoSRN^+^ [Ours]		53.35/0.9991	46.87/0.9960	44.02/0.9926	42.16/0.9890	40.68/0.9847	39.63/0.9806

## Data Availability

Data are contained within the article.
